# Investigation of the effects of different processing methods on the selected nutritional properties of pumpkin and determining the appropriate process for pumpkin yogurt

**DOI:** 10.1002/fsn3.3580

**Published:** 2023-07-28

**Authors:** Hatice Sıçramaz, Ahmet Ayar

**Affiliations:** ^1^ Department of Food Engineering, Faculty of Engineering Sakarya University Sakarya Turkey

**Keywords:** *Cucurbita*, nutritional properties, preprocessing, pumpkin, yogurt

## Abstract

The processing methods, especially cooking, can cause quality losses, particularly in the nutritional value of the fresh product. This study investigated the effects of preprocessing methods on the nutritional properties of pumpkin and the physicochemical and sensory properties of pumpkin yogurt. Two different pumpkin varieties (*Cucurbita pepo* and *Cucurbita maxima*) were subjected to three different preprocessing methods (freeze‐drying, boiling, and baking). Boiling significantly increased antioxidant activity (*p* ≤ .05), followed by baking. *C. maxima* had higher TDF and TPC than *C. pepo*, but in both pumpkin varieties, TDF did not change with heat treatment (boiling and baking), while TPC decreased. Mineral contents remained the same or decreased with heat treatment, except for Mn and Fe. In particular, the addition of *C. maxima* significantly affected the color parameters (L*, a*, b*) of yogurt and improved WHC (from 68.9% to 91.6%) and hardness (from 58.0 to 193.5 g; *p* ≤ .05). The sensory evaluation concluded that heat‐treated (boiled and baked) samples were preferred more than freeze‐dried raw ones. In conclusion, the results revealed that adding boiled and baked pumpkins, especially the preference for *C. maxima* instead of *C. pepo*, improved the quality parameters of yogurt.

## Graphical abstract

Our manuscript aimed to investigate the nutritional changes after pumpkin cooking applications. In addition, yogurt fortification with the pumpkins was observed in terms of sensory, textural, and physicochemical properties of the products. The results will provide know‐how for pumpkin yogurt production with higher nutritive values and better product quality.

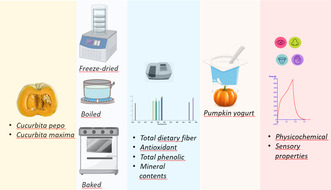

## 1 INTRODUCTION

Pumpkin is a widely consumed nutritious vegetable from the *Cucurbitaceae* family, which is cultivated all over the world. It is regarded as a medicinal plant owing to its nutritional constituents and their health benefits against many diseases (Hussain et al., 2022; Wang et al., 2012). Dietary polyphenols reduce neurological diseases via regulation of microbiota–gut–brain functions (Li et al., 2022). In addition to being a good source of vitamins and minerals, it is also a rich source of dietary fiber, antioxidants, and bioactive compounds (Dhiman et al., 2009). Due to its versatility, pumpkin is used as an ingredient or flavoring agent in various food products, including soups, stews, and pies.

The fortification of yogurt with functional foods is an emerging aspect of food science (Ahmad et al., 2022). One promising area of pumpkin utilization in a human diet is its addition to flavored yogurts as a functional food. Flavored yogurts are typically made by adding fruit puree or jam to plain yogurt (Codex Alimentarius, 2018). On the other hand, the processes applied in puree production can change the nutritional values of the vegetable (Kaur et al., 2019). Examining the effects of processing types on nutritional properties and deciding on the cooking method according to the characteristics of the final product is an important research subject in food science.

Therefore, the objective of this study was to evaluate the effects of preprocessing methods (freeze‐drying, boiling, and baking) on the nutritional properties (dietary fiber, bioactive substances, and mineral content) of two pumpkin varieties (*Cucurbita pepo* and *Cucurbita maxima*) and the effects of the preprocessing on the physicochemical and sensory properties of pumpkin yogurt.

## 2 MATERIALS AND METHODS

### 2.1 Supply and preprocessing of pumpkins

Thirty kilogram of pumpkin for each variety was obtained from the agricultural producers of Sakarya. After washing under water, the seeds and skins were removed. The remaining flesh was cut into 2 cm^3^ pieces and processed using three methods. In the freeze‐drying method, the cubes were freeze‐dried using Labconco Freezone 6 freeze dryer at −45°C under a vacuum of 0.045 mbar for 6 days. In the boiling method, drinking water was added to pumpkins at a ratio of 1:1 (w:w), then boiled for 1.5 h on a 350°C heater. In the baking method, the pumpkin cubes were cooked in the oven (Siemens iQ700) at 150°C for 70 min. All processed groups were then separately homogenized with a blender. The freeze‐dried raw pumpkins as powder and the boiled and baked pumpkins as puree were obtained. Finally, the processed pumpkins were used in the production of flavored yogurts.

### 2.2 Production of yogurts

For instance, plain yogurt was produced to be used in all sample groups. UHT whole cow's milk (3.0% fat and 3.0% protein) was inoculated with a mixed culture of *S. thermophilus* and *L. delbrueckii subsp. bulgaricus* (CHR Hansen YC‐350 yogurt culture) and incubated at 43°C until the pH of the milk reached 4.6. After the incubation was completed, the plain yogurt was mixed, and the clot was broken. Then, it was split into lots, and other ingredients were added according to the formulations given in Table 1. The pumpkin and sugar concentrations in manufacturing were determined by preliminary sensory tests prepared with at least five different concentrations. Plain and flavored yogurt productions were duplicated by the procedure described in this section. The samples were kept at 4°C until the analysis.

**TABLE 1:** The control and pumpkin yogurt formulations.

	Yogurt (g)	Sugar (g)	*Cucurbita pepo* (g)	*Cucurbita maxima* (g)	Pretreatment of *pumpkin* species
Control yogurts					
NC	100	‐	‐	‐	‐
PC	95	5	‐	‐	‐
Freeze‐dried raw pumpkin‐added yogurts					
R35P	91.5	5	3.5	‐	Raw, freeze‐dried
R35M	91.5	5	‐	3.5	Raw, freeze‐dried
R50P	90	5	5.0	‐	Raw, freeze‐dried
R50M	90	5	‐	5.0	Raw, freeze‐dried
Boiled pumpkin‐added yogurts					
B250P	70	5	25.0	‐	Boiled in hot water
B250M	70	5	‐	25.0	Boiled in hot water
B300P	65	5	30.0	‐	Boiled in hot water
B300M	65	5	‐	30.0	Boiled in hot water
Baked pumpkin‐added yogurts					
O250P	70	5	25.0	‐	Baked in the oven
O250M	70	5	‐	25.0	Baked in the oven
O300P	65	5	30.0	‐	Baked in the oven
O300M	65	5	‐	30.0	Baked in the oven

*Note*: Product codes of NC and PC: control samples. R, B, O: raw, boiled, baked, respectively. Numerical value: concentration (i.e., 35: 3.5%, 250: 25%). P and M: *C. pepo* and *C. maxima*, respectively.

The characteristics of pumpkins and yogurts were determined by at least two replicate analyses.

### 2.3 Nutritive properties of pumpkin samples

Raw and preprocessed (boiled or baked) pumpkin samples to be used to determine nutritional components were freeze‐dried before analysis. The total dietary fiber (TDF) of pumpkins was determined following the Sigma‐Aldrich protocol provided in the TDF‐100A Kit (Sigma‐Aldrich Inc.), and the results were given as % in dry matter (DM). For analysis of bioactive components, extracts were prepared by mixing 0.5 g of dried sample in 10 mL of 70% methanol using a homogenizer. The mixture was kept in an ultrasonic water bath at 20°C for 15 min and then centrifuged at 1500 *g* at 4°C for 10 min. After separating the supernatant, the remaining part was used as an extract in the analyses. The total phenolic compound (TPC) analysis was carried out according to Cerit et al. (2016). In the method, 100 μL of the extract was mixed with 0.2 mL of Folin–Ciocalteu reagent and 2 mL of distilled water. The mixture was incubated for 3 min at room temperature. Then, 1 mL of 20% sodium carbonate was added and kept in the dark at room temperature for 1 h. Absorbance was measured at 765 nm using a Shimadzu UV‐1240 spectrophotometer. The results were given as mg of gallic acid equivalent (GAE) per 100 g of DM. The antioxidant capacity was determined in terms of DPPH (2,2‐diphenyl‐1‐picrylhydrazyl) radical scavenging activity with some modifications of the method recommended by Shaterabadi et al. (2020). The DPPH solution was prepared at a final concentration of 0.05 mM by dissolving in 100% methanol. Three milliliter of DPPH solution was added to 200 μL of extract. After being kept in the dark for 30 min, absorbance was measured at 517 nm using a spectrophotometer (Schimadzu UV mini‐1240). The DPPH scavenging activity was obtained as Trolox equivalent using a calibration curve.

Concentrations of eight elements (Ca, Cu, Fe, K, Mg, Mn, P, and Zn) were determined after freeze‐drying the untreated and cooked pumpkin samples. 0.5 g of sample was digested using a microwave (MARS‐5 CEM) in the presence of nitric acid and hydrogen peroxide. The mineral contents were analyzed using an inductively coupled plasma‐atomic emission spectrometer (ICP‐MS, Agilent 7700 series; Agilent Technologies) as described by Aktaş et al. (2015).

### 2.4 Physicochemical properties of yogurt samples

The pH and titratable acidity of the yogurt samples were measured on 1, 7, and 14 days of storage. The pH was analyzed at 25°C using a pH meter (WTW 720). The titratable acidity was determined in terms of lactic acid percent (LA%) according to the AOAC methods (AOAC, 2000). The DM, protein, and ash content of the yogurts were analyzed after a week of storage using the official methods (AOAC, 2000). The textural properties of yogurts were observed using a texture analyzer (Brookfield CT3, Brookfield Engineering Laboratories). In the analysis, a compression test was applied using TA 4/1000 cylindrical probe to a distance of 20 mm with 4.0 g of trigger force and 1 mm/s test speed, and the results were expressed in terms of hardness (g). The water holding capacity (WHC) of yogurts was measured using the method described by Gomes et al. (2023) with some modifications: Approximately 20 g of yogurt sample was centrifuged at 1250 *g* at 4°C for 10 min. The WHC was expressed as the percentage ratio of the mass remaining after serum separation to the initial yogurt weight. The colors of yogurts were determined using a tintometer (Lovibond RT300) and expressed in CIELAB system where L* indicated lightness, a* redness or greenness, and b* yellowness or blueness.

The sensory properties were measured by an internationally accepted and widely used 9‐point hedonic scale (Nicolas et al., 2010). The panelists were trained males and females aged between 18 and 45, consisting of 10 students and staff of Sakarya University (Turkey). For the evaluations of color, texture, taste, and general acceptance, responses were recorded, indicating 1 = dislike extremely and 9 = like extremely. The flavor intensity and sweetness parameters were asked for rating as 1 = none and 9 = over.

### 2.5 Statistical analyses

Data of two production replicates with twice analysis were initially subjected to analysis of variance (ANOVA) using Minitab 16 software (Minitab Inc.). Results were compared using Tukey's test with a significance level of 5%. Multivariate functional principal component analysis (PCA) was applied for further evaluation of mineral content and sensory data.

## 3 RESULTS AND DISCUSSION

### 3.1 Total dietary fiber and bioactive contents of processed pumpkins

Raw flesh DM of *C. pepo* and *C. maxima* were 7.55% and 8.04, respectively, and increased to 94.5–95.0 by freeze‐drying. According to Table 2, the baking process increased DM more than boiling, probably due to evaporation. The fiber content and bioactive materials of the processed pumpkins were measured after freeze‐drying to eliminate the moisture effect, and the results are given in Table 2. The TDF content was not affected by the process type (*p* > .05). The TDF amount of *C. maxima* (34.4% in DM) was higher than *C. pepo* (24.3% in DM) in all processed groups. On the other hand, Kim et al. (2012) reported TDF amounts considerably lower than in our study and reported TDF of *C. pepo* as higher than *C. maxima* (10.94% and 6.44% in DM, respectively), contrary to our findings. Mehditabar et al. (2020) reported the water‐soluble and ‐insoluble fiber fractions of baked pumpkin as 14.13 and 19.56 g per 100 g DM, respectively, with a higher total fiber amount than that in our study. According to the values in the USDA National Nutrient Database (USDA, 2018), pumpkin contains 2.9% dietary fiber. When our results were reflected in the total weight (including moisture content), comparable results were observed. Our results were obtained in the range 2.4%–3.1% for *C. pepo* and 2.9%–4.1% for *C. maxima*, considering the moisture ratios.

**TABLE 2:** The DM, TDF, antioxidant capacity, and TPC results of processed pumpkins.

Pumpkin variety	Treatment	DM	TDF	DPPH scavenging activity	TPC
*Cucurbita pepo*	Freeze‐dried, raw	95.0 ± 0.2 A	24.3 ± 0.8 B	758.9 ± 11.4 C	172.9 ± 20.0 B
	Boiled	10.1 ± 0.0 B	24.2 ± 1.0 B	2019.6 ± 268.9 A	42.2 ± 10.1 D
	Baked	12.6 ± 1.0 C	24.3 ± 4.0 B	1354.7 ± 32.5 B	42.6 ± 2.9 D
*Cucurbita maxima*	Freeze‐dried, raw	94.5 ± 0.5 A	34.4 ± 0.4 A	501.2 ± 32.8 C	244.5 ± 29.1 A
	Boiled	8.2 ± 0.1 BC	35.6 ± 2.9 A	2093.2 ± 109.6 A	90.2 ± 8.8 C
	Baked	11.6 ± 0.0 D	35.2 ± 0.3 A	1503.2 ± 56.9 B	73.3 ± 9.8 CD

*Note*: Different capital letters in the same column represent significant differences between samples (*p* ≤ .05).

Abbreviations: DM, dry matter%; TDF, total dietary fiber % in dry matter; DPPH scavenging activity, μmol Trolox/100 g dry matter; TPC, mg gallic acid equivalent in 100 g dry matter.

The highest amount of antioxidant substance was determined in the boiled groups, then the baked ones, and the least was in the raw materials. In conclusion, the most effective method to increase antioxidant capacity was boiling, probably due to cell lysis. There are studies in the literature supporting that cell lysis increases DPPH scavenging activity (Barba et al., 2015). Similarly, in a study examining the physicochemical and antioxidant properties of *C. moschata*, the DPPH amount increased from 77 to 99 mg GAE/100 g DM after drying in a hot‐air tray dryer (Promsakha na Sakon Nakhon et al., 2017). In a study investigating the effects of cooking techniques on the nutritional properties of pumpkin leaves, it was reported that steam application reduces antioxidant loss compared to the boiling process (Mashiane et al., 2021).

It was also determined in our present study that *C. maxima* was richer in TPC than *C. pepo*. However, the amount of TPC was significantly reduced by boiling and baking compared to the raw product in both varieties (*p* ≤ .05). Regardless of the cooking method, the TPC content of *C. pepo* decreased from 172.9 to 42.2–42.6, and *C. maxima* decreased from 244.5 to 73.3–90.2 mg GAE/100 g DM. Temperature applications are known to damage TPC (Vega‐Gálvez et al., 2009). Malakar and Arora (2022) developed a solar dryer to dry pumpkin slices and increased the amount of TPC from 8.63 to 17.44 mg GAE/g DM in the new system.

### 3.2 Mineral contents of processed pumpkins

The mineral contents of pumpkins are given in Table 3. Among the mineral substances, potassium (K) was the major mineral of pumpkins, ranging from 25.13 to 29.65 g in 100 g of DM. It was followed by Ca, P, and Mg. While the amounts of K, Ca, P, and Mg were not affected by heat treatments in *C. maxima*, a reduction was observed in *C. pepo*. In a study on the nutritive values of pumpkin leaves, it was reported that the amount of K remained the same with boiling in water but slightly decreased with steam blanching (Sunmonu et al., 2021). The Fe and Mn contents we examined did not decrease with heat treatment; on the contrary, they remained the same or increased. While Cu was resistant to boiling in *C. maxima*, in *C. pepo* it was damaged by both of the heat treatments. The Zn decreased by boiling in *C. pepo*, but remained the same in *C. maxima*. Badr et al. (2011) reported a Zn value of 320.5 mg/100 g DM in the pumpkin they analyzed, which is considerably higher than that in our study. On the other hand, Ca, in the same study, which was reported as 3.67 g, and Fe and Cu minerals, which were reported as 91.33 and 16.25 mg per 100 g dry basis, respectively, were closer to the values in our study. Vidhya et al. (2022), on the other hand, found the Zn content of *C. maxima* and *C. pepo* as 7.4 and 1.3 mg, respectively, per 100 g of dry basis of pumpkin flesh. Especially in the amount of Zn, remarkable differences were detected in the literature. The relationship between the mineral content in the soil or fertilizer and its reflection in the product should be investigated by agricultural experts.

**TABLE 3:** The mineral content of processed pumpkins.

Pumpkin variety	*Cucurbita pepo*			*Cucurbita maxima*		
Treatment	Raw	Boiled	Baked	Raw	Boiled	Baked
Minerals*. g/100 g dry matter						
K*	29.65 ± 0.21 A	25.13 ± 0.24 D	25.29 ± 0.19 D	28.06 ± 0.22 BC	27.62 ± 0.26 C	28.74 ± 0.18 B
Ca*	6.55 ± 0.22 A	4.24 ± 0.14 C	4.17 ± 0.15 C	6.03 ± 0.19 AB	6.38 ± 0.21 AB	5.76 ± 0.21 B
P*	6.48 ± 0.09 A	3.74 ± 0.05 C	3.74 ± 0.04 C	5.94 ± 0.08 B	5.76 ± 0.08 B	6.04 ± 0.08 B
Mg*	2.46 ± 0.06 A	2.13 ± 0.12 AB	2.00 ± 0.08 B	2.15 ± 0.07 AB	2.31 ± 0.10 AB	2.21 ± 0.08 AB
Minerals. mg/100 g dry matter						
Fe	72.9 ± 8.6 BC	102.6 ± 8.1 AB	68.7 ± 8.4 BC	62.8 ± 8.5 C	73.9 ± 8.2 BC	115.3 ± 10.2 A
Zn	27.1 ± 1.6 A	16.8 ± 3.5 B	24.1 ± 2.8 AB	26.8 ± 1.7 AB	30.9 ± 2.5 A	23.0 ± 2.4 AB
Cu	15.1 ± 0.9 B	9.9 ± 0.6 C	10.1 ± 0.8 C	18.6 ± 0.4 A	15.7 ± 1.0 AB	13.6 ± 0.8 B
Mn	4.76 ± 1.30 BC	8.27 ± 0.61 AB	8.40 ± 0.88 A	4.21 ± 1.23 C	4.85 ± 0.54 ABC	4.63 ± 0.49 C

*Note*: Different capital letters in the same line represent significant differences between samples (*p* ≤ .05).

**FIGURE 1:**
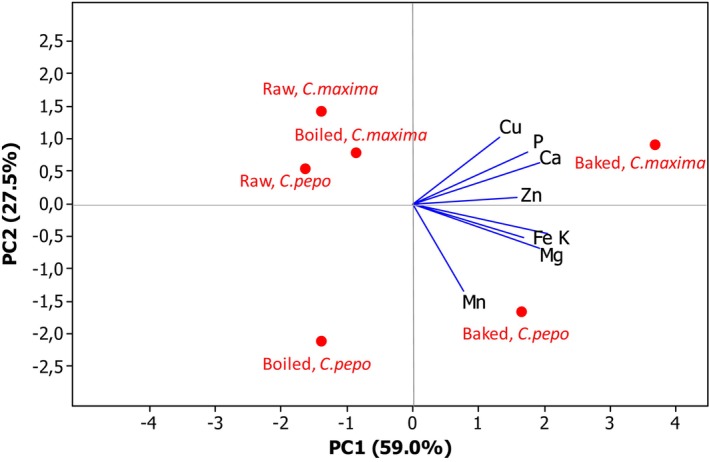
The PCA of the mineral content of processed pumpkins.

When the mineral contents were examined by PCA analysis, two distinct groups—PC1 and PC2—were observed (Figure 1). According to PC1, the baked products of both pumpkins were in the positive area, and the raw and boiled types were in the negative area. The baked *C. maxima* in the positive area were dominant in Zn, Ca, P, and Cu, while baked *C. pepo* could be represented by the other minerals—Mn, Mg, Fe, and K. According to PC2, all processed products of *C. maxima* and the raw group of *C. pepo* were in the positive area, while the boiled and baked versions of *C. pepo* were located in the negative area.

### 3.3 Physicochemical properties of yogurts incorporated with processed pumpkins

The pH and acidity values of yogurt samples for 14 days of storage are given in Table 4. The addition of pumpkin increased the pH in all products compared to the control. The acidity was not affected in the freeze‐dried raw pumpkin‐added products, but decreased in boiled and baked groups. Freeze‐dried raw pumpkin‐added yogurts were also better protected against pH decrease and acidity increase during storage compared to the control and the other samples. When evaluated together with the DM values shown in Table 5, it was determined that there might be a relationship between the progress of pH and DM. In yogurt with higher DM, the pH decrease was also slower, probably due to a decrease in free water for microbial and chemical activities.

**TABLE 4:** Variation in the pH and acidity values of different yogurt formulations on days 1, 7, and 14.

Product code	pH						Titratable acidity (LA%)					
	Day 0		Day 7		Day 14		Day 0		Day 7		Day 14	
Control yogurts												
NC	4.40 ± 0.00	G a	4.35 ± 0.01	F b	4.25 ± 0.00	F c	1.15 ± 0.00	B c	1.20 ± 0.00	B b	1.22 ± 0.01	B a
PC	4.38 ± 0.01	G a	4.29 ± 0.00	G b	4.18 ± 0.01	G c	1.09 ± 0.01	B b	1.12 ± 0.01	C b	1.25 ± 0.02	AB a
Freeze‐dried raw pumpkin‐added yogurts												
R35P	4.50 ± 0.01	DE a	4.48 ± 0.01	C a	4.40 ± 0.01	CD b	1.17 ± 0.02	B a	1.21 ± 0.01	AB a	1.28 ± 0.08	AB a
R35M	4.51 ± 0.01	D a	4.52 ± 0.01	B a	4.42 ± 0.01	C b	1.16 ± 0.00	B b	1.24 ± 0.02	AB a	1.25 ± 0.01	AB a
R50P	4.58 ± 0.01	B a	4.58 ± 0.01	A a	4.47 ± 0.01	B b	1.17 ± 0.01	B c	1.26 ± 0.00	A b	1.30 ± 0.01	AB a
R50M	4.58 ± 0.01	B a	4.59 ± 0.01	A a	4.51 ± 0.01	A b	1.28 ± 0.02	A ab	1.23 ± 0.00	AB b	1.33 ± 0.01	A a
Boiled pumpkin‐added yogurts												
B250P	4.47 ± 0.01	EF a	4.26 ± 0.01	H b	4.17 ± 0.00	G c	0.86 ± 0.00	CD b	0.94 ± 0.02	DEF a	0.94 ± 0.00	CDE a
B250M	4.46 ± 0.00	F a	4.29 ± 0.01	G b	4.18 ± 0.00	G c	0.93 ± 0.01	C b	0.95 ± 0.00	DEF b	1.02 ± 0.01	CD a
B300P	4.54 ± 0.01	CD a	4.47 ± 0.01	CD b	4.38 ± 0.01	D c	0.75 ± 0.08	E a	0.88 ± 0.02	G a	0.90 ± 0.01	E a
B300M	4.51 ± 0.02	DE ab	4.53 ± 0.01	B a	4.45 ± 0.00	B b	0.86 ± 0.00	CD c	0.93 ± 0.00	EFG b	0.95 ± 0.01	CDE a
Baked pumpkin‐added yogurts												
O250P	4.52 ± 0.01	D a	4.40 ± 0.00	E b	4.30 ± 0.01	E c	0.90 ± 0.00	CD a	0.96 ± 0.03	DEF a	0.92 ± 0.00	DE a
O250M	4.53 ± 0.01	D a	4.39 ± 0.00	E b	4.33 ± 0.01	E c	0.91 ± 0.00	CD b	0.98 ± 0.01	D a	1.02 ± 0.01	C a
O300P	4.57 ± 0.01	BC a	4.44 ± 0.01	D b	4.32 ± 0.01	E c	0.83 ± 0.00	DE c	0.91 ± 0.00	FG b	0.92 ± 0.00	DE a
O300M	4.62 ± 0.00	A EGG	4.53 ± 0.01	B b	4.40 ± 0.00	CD c	0.87 ± 0.00	CD c	0.97 ± 0.00	DE b	0.99 ± 0.01	CDE a

*Note*: Different capital letters in the same column represent significant differences between samples in the same storage time, and the small letters in the same line represent significant differences in the property during the storage time. Product codes of NC and PC: control samples. R, B, O: raw, boiled, baked, respectively. Numerical value: concentration (i.e., 35: 3.5%, 250: 25%). P and M: C. pepo and C. maxima, respectively.

**TABLE 5:** The general physicochemical properties of yogurt samples on day 1.

Product code	DM (%)	Protein (%)	Ash (%)	Hardness (g)	WHC (%)	L*	a*	b*
Control yogurts								
NC	11.4 ± 0.02 I	2.86 ± 0.11 BC	0.73 ± 0.05 EF	85.5 ± 5.7 EF	60.7 ± 0.1 J	78.7 ± 0.1 A	−1.41 ± 0.00 E	4.38 ± 0.12 D
PC	16.9 ± 0.02 D	2.43 ± 0.13 CDE	0.71 ± 0.00 EF	58.0 ± 1.4 GHI	68.9 ± 0.2 FG	77.2 ± 0.2 A	−1.48 ± 0.01 E	3.86 ± 0.06 D
Freeze‐dried raw pumpkin‐added yogurts								
R35P	20.1 ± 0.04 B	3.02 ± 0.13 AB	0.98 ± 0.01 C	100.0 ± 2.1 DE	61.6 ± 0.4 IJ	66.2 ± 2.4 CDEF	−1.39 ± 0.08 E	8.49 ± 2.46 CD
R35M	20.2 ± 0.01 B	3.35 ± 0.17 A	1.00 ± 0.01 BC	168.0 ± 7.8 B	80.5 ± 0.5 C	65.4 ± 1.2 CDEF	2.19 ± 1.56 CD	20.38 ± 2.52 B
R50P	21.5 ± 0.03 A	2.89 ± 0.16 ABC	1.08 ± 0.02 AB	120.3 ± 5.3 C	67.7 ± 0.7 FG	64.8 ± 0.5 DEF	−1.40 ± 0.08 E	12.18 ± 1.52 C
R50M	21.5 ± 0.08 A	3.13 ± 0.07 AB	1.13 ± 0.05 A	193.5 ± 9.9 A	91.6 ± 0.6 A	69.2 ± 0.1 BCD	9.48 ± 0.11 A	33.53 ± 0.06 A
Boiled pumpkin‐added yogurts								
B250P	16.5 ± 0.06 E	2.19 ± 0.10 DE	0.69 ± 0.01 EF	51.0 ± 0.7 HI	66.5 ± 0.5 GH	63.4 ± 2.4 EF	−1.55 ± 0.19 E	8.26 ± 1.83 CD
B250M	16.3 ± 0.01 F	2.45 ± 0.12 CDE	0.72 ± 0.03 EF	67.5 ± 2.1 FGH	72.6 ± 0.6 DE	67.9 ± 0.0 CDE	3.05 ± 0.04 C	25.49 ± 0.88 B
B300P	16.1 ± 0.02 G	2.02 ± 0.14 E	0.66 ± 0.03 F	46.5 ± 0.7 I	64.4 ± 0.4 HI	72.4 ± 0.0 B	0.84 ± 0.04 D	21.78 ± 0.26 B
B300M	15.8 ± 0.05 H	2.25 ± 0.08 DE	0.72 ± 0.01 EF	75.8 ± 1.1 FG	74.5 ± 0.5 D	69.6 ± 0.2 BC	6.25 ± 0.03 B	32.64 ± 0.42 A
Baked pumpkin‐added yogurts								
O250P	17.3 ± 0.03 C	2.25 ± 0.04 DE	0.76 ± 0.01 DE	57.3 ± 3.9 HI	69.8 ± 0.8 EF	63.0 ± 2.1 F	−1.59 ± 0.11 E	11.66 ± 2.39 C
O250M	16.6 ± 0.02 E	2.51 ± 0.16 CD	0.76 ± 0.01 DE	118.3 ± 5.3 C	82.6 ± 0.6 C	69.4 ± 0.1 BC	6.17 ± 0.34 B	32.26 ± 0.21 A
O300P	16.6 ± 0.02 E	2.07 ± 0.08 DE	0.73 ± 0.01 EF	64.3 ± 3.2 GHI	70.0 ± 0.0 EF	62.7 ± 0.1 F	−1.77 ± 0.03 E	12.90 ± 0.02 C
O300M	17.0 ± 0.03 D	2.20 ± 0.09 DE	0.83 ± 0.03 D	115.5 ± 2.1 CD	87.6 ± 0.6 B	68.6 ± 0.1 BCD	8.92 ± 0.37 A	36.72 ± 0.17 A

*Note*: Different capital letters in the same column represent significant differences between samples (*p* ≤ .05). Product codes of NC and PC: control samples. R, B, O: raw, boiled, baked, respectively. Numerical value: concentration (i.e., 35: 3.5%, 250: 25%). P and M: *C. pepo* and *C. maxima*, respectively.

Abbreviations: DM, Dry matter; WHC, Water holding capacity; L*, a*, b*, color properties.

The other analyzed physicochemical properties of yogurts are given in Table 5. Sugar addition increased the DM of control, as expected. The DM values of freeze‐dried raw pumpkin‐added yogurts increased with the increasing amount of pumpkin and were significantly higher than the control, regardless of the pumpkin variety. In general, the DM of *C. pepo‐*added yogurts was higher than *C. maxima*‐added ones. When the protein was analyzed in total mass, higher amounts were encountered in freeze‐dried raw products, probably because of higher DM. It was also observed that the change in pumpkin variety did not affect the protein ratios. Total ash was high in raw material‐added yogurts and was similar to the control in other products. The DM increase in freeze‐dried raw material‐added yogurts also increased the hardness of the products. Sugar addition significantly reduced the hardness from 85.5 to 58.0 g in control samples. The boiled and baked pumpkin‐added groups had hardness values similar to the PC. It was also determined that the hardness of the yogurts with *C. maxima* was higher than those with *C. pepo*. Contrary to our results, Yildiz and Ozcan (2019) reported a decrease in the hardness of yogurt with the addition of pumpkin puree baked at 85–90°C for 15 min, probably as a result of the presence of higher moisture in the final product due to the lower thermal processing than applied in our study. As observed in all yogurt groups of our present study, the WHC considerably increased with the addition of *C. maxima* compared to *C. pepo*. In addition, it was determined that the WHC values were independent of the DM. Similarly, Bakirci et al. (2017) measured the lowest WHC in control yogurt and reported an increase with increasing pumpkin concentration. When the color properties were examined in terms of L*, a*, and b*, the L* value decreased in all pumpkin‐added products compared to control samples. When the differences between *C. maxima* and *C. pepo* were evaluated, the L* values were measured as similar or higher in *C. maxima*‐added products. However, the variety of pumpkins significantly affected a* and b* values. The a* values of yogurts containing *C. pepo* were similar to the control samples, which were measured as −1.4 (in green area), while the value was between +2.2 and +9.5 (in red area) in products containing *C. maxima*. In addition, the a* value of *C. maxima*‐added products increased significantly with increasing pumpkin concentration. Similarly, b* values were in the direction of yellowness in all products, but adding *C. pepo* revealed similar results to the control, while *C. maxima* raised this value significantly. Bakirci et al. (2017) obtained color values in the yogurts with *C. moschata*, similar to our findings with *C. maxima*.

### 3.4 Sensory properties of yogurts with processed pumpkins

Yogurts were evaluated according to color, texture, taste, flavor intensity, sweetness, and general acceptance, and the results are given in Table 6. While *C. pepo* was not liked in the color evaluation, *C. maxima* was scored similarly to the control product. The R50M and B300P products (containing 5% freeze‐dried raw *C. maxima* and 30% boiled *C. pepo*, respectively) received the lowest scores in texture evaluation. The O300M and O250M products, including baked *C. maxima*, were scored as the most demanded products and revealed comparable scores to the control product. According to the taste evaluation, freeze‐dried raw pumpkins were not liked. Boiled and baked products of *C. pepo* and *C. maxima* were scored similarly to each other and the control sample. Flavor intensity was felt at the same level in all pumpkin yogurts. The negative control (NC) yogurt had the lowest sweetness score as it contained no added sugar. The positive control (PC) got the highest sweetness score in all groups. Although all yogurt groups except for NC had the same amount of sugar, the freeze‐dried raw pumpkin addition reduced the sweetness intensity. The addition of *C. maxima* variety of pumpkin also reduced sweetness, while *C. pepo* revealed comparable results to the PC. R50P was not appreciated in general acceptance, but other products received similar scores to the control yogurts. Similarly, in a study examining the properties of stirred pumpkin yogurts, the addition of *C. moschata*, *C. maxima*, and *C. pepo* resulted in similar sensory properties to the control yogurts (Barakat & Hassan, 2017).

**TABLE 6:** The sensory properties of yogurt samples.

Product code	Color	Texture	Taste	Flavor intensity	Sweetness	General acceptance
Control yogurts						
NC	8.6 ± 1.0 AB	7.4 ± 0.9 AB	7.1 ± 1.3 A	1.0 ± 0.0 B	1.0 ± 0.0 D	7.0 ± 1.3 A
PC	8.7 ± 0.7 A	6.4 ± 1.1 BCD	7.0 ± 1.2 A	1.0 ± 0.0 B	6.1 ± 1.3 A	7.0 ± 0.9 A
Freeze‐dried raw pumpkin‐added yogurts						
R35P	5.0 ± 0.8 F	5.6 ± 1.5 BCD	4.5 ± 1.6 C	4.8 ± 1.5 A	5.1 ± 0.3 AB	5.3 ± 1.2 AB
R35M	7.7 ± 1.1 ABCD	6.0 ± 1.2 ABCD	5.9 ± 1.4 ABC	6.1 ± 1.5 A	3.8 ± 0.8 C	6.0 ± 1.3 AB
R50P	6.0 ± 1.2 DEF	5.6 ± 1.4 BCD	4.9 ± 1.1 BC	5.1 ± 1.2 A	4.5 ± 0.9 BC	5.0 ± 0.8 B
R50M	6.8 ± 1.4 BCDEF	4.5 ± 1.4 D	4.7 ± 1.1 BC	5.6 ± 1.4 A	4.1 ± 0.7 BC	5.3 ± 1.7 AB
Boiled pumpkin‐added yogurts						
B250P	5.8 ± 1.6 EF	5.7 ± 0.9 BCD	5.6 ± 1.6 ABC	4.9 ± 1.0 A	5.1 ± 0.6 AC	6.2 ± 0.9 AB
B250M	7.4 ± 1.0 ABCDE	6.6 ± 1.4 ABC	5.8 ± 1.2 ABC	5.1 ± 0.7 A	4.9 ± 0.9 BC	6.7 ± 0.9 AB
B300P	6.4 ± 1.2 CDEF	5.1 ± 1.4 CD	5.7 ± 1.3 ABC	5.2 ± 1.0 A	5.3 ± 1.0 AC	5.9 ± 1.0 AB
B300M	7.6 ± 0.7 ABCD	6.3 ± 1.6 ABCD	5.6 ± 1.2 ABC	5.3 ± 1.1 A	4.9 ± 0.7 BC	5.8 ± 0.7 AB
Baked pumpkin‐added yogurts						
O250P	6.7 ± 1.6 CDEF	5.7 ± 1.5 BCD	6.5 ± 1.4 AB	5.2 ± 1.1 A	5.1 ± 0.9 AB	6.3 ± 1.5 AB
O250M	7.7 ± 1.2 ABCD	7.3 ± 1.3 AB	5.7 ± 1.1 ABC	5.3 ± 1.2 A	4.2 ± 0.8 BC	5.4 ± 1.0 AB
O300P	6.9 ± 1.6 ABCDE	6.1 ± 1.2 ABCD	6.5 ± 1.2 AB	5.4 ± 1.2 A	5.1 ± 0.6 AB	6.1 ± 1.0 AB
O300M	8.2 ± 0.9 ABC	7.8 ± 1.2 A	6.1 ± 1.2 ABC	6.1 ± 1.1 A	4.5 ± 0.5 BC	6.6 ± 1.2 AB

*Note*: Different capital letters in the same column represent significant differences between samples (*p* ≤ .05). Product codes of NC and PC: control samples. R, B, O: raw, boiled, baked, respectively. Numerical value: concentration (i.e., 35: 3.5%, 250: 25%). P and M: *C*. *pepo* and *C. maxima*, respectively.

**FIGURE 2:**
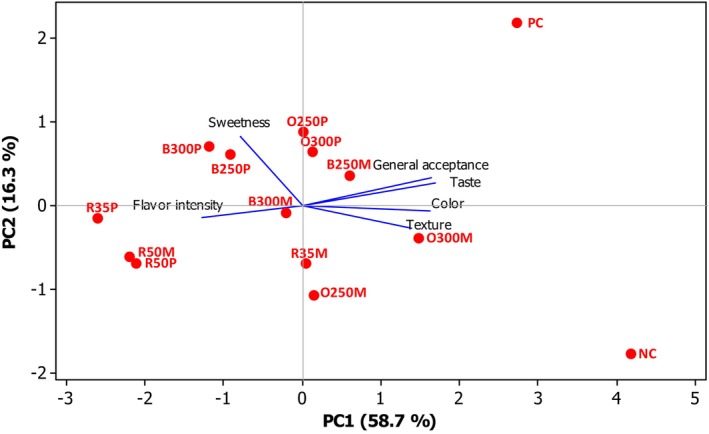
The PCA of the sensory properties of processed pumpkins. Product codes of NC and PC: control samples. R, B, O: raw, boiled, baked, respectively. Numerical value: concentration (i.e., 35: 3.5%, 250: 25%). P and M: *C. pepo* and *C. maxima*, respectively.

When the sensory data were analyzed by PCA (Figure 2), *C. pepo* was found to be distinctive for sweetness. It was observed that the products made with freeze‐dried raw materials were not liked in terms of general acceptance and taste. In terms of texture, the baked *C. maxima* yogurts (O250M and O300M) were attributed and similar to the NC. In contrast, the baked batches of *C. pepo* (coded as O250P and O300P) scored lower according to the PCA analysis. Considering the launch of the product to the market, it is recommended to first taste it to a wider panel group.

## 4 CONCLUSION

This study was carried out to determine the preprocessing conditions, variety, and concentrations of pumpkin required to obtain pumpkin yogurt with enriched nutritional value and advanced physicochemical and sensory properties. For this purpose, two different pumpkin varieties were subjected to three different pretreatments before they were added to the yogurt. According to nutritional values, incorporating *C. maxima* in yogurt was recommended instead of *C. pepo*. The *C. maxima* was also more appropriate when yogurts were evaluated for hardness, WHC, and color. Furthermore, evaluation of pretreatments of pumpkins revealed that boiling and baking increased antioxidant capacity and resulted in higher sensory scores when added to yogurt. In conclusion, *C. maxima* in baked form was recommended to be used in pumpkin yogurt. Future studies should focus on the effects of these parameters on bioavailability.

## AUTHOR CONTRIBUTIONS

**Hatice Sıçramaz:** Conceptualization (equal); data curation (equal); formal analysis (lead); investigation (equal); methodology (equal); writing – original draft (lead); writing – review and editing (equal). **Ahmet Ayar:** Conceptualization (equal); data curation (equal); funding acquisition (lead); investigation (equal); methodology (equal); project administration (lead); supervision (lead); writing – review and editing (equal).

## CONFLICT OF INTEREST STATEMENT

The authors declare no conflict of interest.

## Data Availability

The data that support the findings of this study are available from the corresponding author upon reasonable request.
